# Association between infant mortality and parental educational level: An analysis of data from Vital Statistics and Census in Japan

**DOI:** 10.1371/journal.pone.0286530

**Published:** 2023-06-14

**Authors:** Tasuku Okui

**Affiliations:** Medical Information Center, Kyushu University Hospital, Fukuoka City, Japan; Walden University, UNITED STATES

## Abstract

This study investigated the association between parental educational level and infant mortality using data from Vital Statistics and Census in Japan. We used the Census data in 2020 and birth and mortality data from the Vital Statistics from 2018 to 2021 in Japan. Data linkage was conducted between birth data and the Census to link the educational level with parents for birth data and between the birth data and mortality data to identify births that resulted in infant mortality. Four educational levels were compared: “junior high school,” “high school,” “technical school or junior college,” and “university.” A multivariate logistic regression model was used to investigate an association between parental educational level and infant mortality using other risk factors as covariates. After the data linkage, data on 890,682 births were analyzed. The proportion of junior high school or high school graduates was higher among fathers and mothers for births with infant mortality compared with that among those for births without infant mortality; in contrast, the proportion of university graduates was lower for births with infant mortality than those without infant mortality. Regression analysis showed that mothers with junior high school or high school graduates were significantly and positively associated with infant mortality compared with those with university graduates. As a conclusion, lower educational level in mothers was positively associated with infant mortality, and it was shown that a difference in infant mortality depending on parental educational level existed in Japan.

## Introduction

Infant mortality is a representative indicator of maternal and child health worldwide, and countries with higher growth of domestic products per capita tend to have a smaller infant mortality rate [1]. Japan has a low infant mortality rate [2], which demonstrates a decreasing trend over the decades and is 1.8 per 1,000 births in 2020 [3]. It is known that reduction of neonatal death by control of diseases is one factor for decrease of infant disease in Japan [4], while it is analyzed that factors other than development of medical technology has also contributed to the reduction [5]. Leppet pointed out factors, such as use of the Maternal and Child Health Handbook, universal access to care, availability of abortion, and high literacy of Japanese citizens as factors for low infant mortality rate in Japan [6]. In contrast, the infant mortality rate varies depending on the characteristics of births, and thus, it is important to identify factors that are associated with a higher infant mortality rate.

Some previous studies have investigated sociodemographic factors associated with infant mortality in Japan [7–11]. Jobless households tend to have a higher infant mortality than the other types of households [10, 11], and non-Japanese mothers have a high risk [8]. In contrast, one study that investigated the association between maternal occupations and infant mortality reported that there were no associations between them [9], and an association between parental socioeconomic status and infant mortality is not fully revealed in Japan. In addition, to the best of our knowledge, no study has investigated the association between parental educational level and infant mortality in Japan. An ecological study showed that proportion of people receiving higher education was negatively associated with infant mortality rate among regions in Japan [12], but studies using individual data for investigating the association has not been conducted in Japan. In other countries, there are some studies investigating an association between parental educational level and infant mortality or child mortality [13–16], and an inequality by parental educational level has been shown. If an association between parental educational level and infant mortality exists in Japan, we might be able to identify key factors associated with infant mortality by investigating differences in health statuses and behaviors depending on parental educational level. In addition, the result will be useful for deciding whether further financial aids are needed for parents or households with lower socioeconomic status as a policy in Japan.

The association between parental educational level and adverse birth outcomes in Japan has been evaluated [17–19], and lower educational level in parents was shown to be associated with higher rates of small-for-gestational age and preterm births. In addition, it was shown from national data that age-adjusted all-cause mortality rate increased as the educational level decreased among men and women in Japan [20]. Therefore, it is possible that infant mortality rate is higher among births from parents with lower educational level in Japan. In this study, we investigated the association between parental educational level and infant mortality using the Vital Statistics and the Census in Japan.

## Materials and methods

### Data

We used individual data for the Census in 2020 and the Vital Statistics from 2018 to 2021 in Japan, and birth data and mortality data were used in the Vital Statistics. The Census is conducted every 5 years, and the educational level is surveyed every 10 years in Japan. We used the Vital Statistics data for multiple years because the number of infant mortality is small in Japan. The Census data were obtained from the Ministry of Internal Affairs and Communications and the Vital Statistics data were obtained from the Ministry of Health, Labor, and Welfare after the approval of data use for the research.

We used information on sex, birth year, birth month, marital status, prefecture, municipality, nationality, and the household’s number in a living area for individuals. Elementary school, junior high school, high school, technical school or junior college, university, and graduate school were available as types of educational levels for graduates.

Information on infant’s sex and birthday, prefecture, municipality, multiplicity of birth, birth order for multiple births, bodyweight, gestational age, legitimacy, birthday and nationality for parents, maternal age, parity, and household occupation were used for birth data. Because we focus on the parental educational level, only legitimate children were used in the analysis. Farmer, self-employed, full-time worker 1, full-time worker 2, others, and jobless are available as a household occupation. Full-time worker 1 means household with a full-time worker working in a company who has less than 100 employees, and full-time worker 2 means a household with a full-time worker working in a company who has 100 or more employees.

In addition, information on infant’s sex and birthday, the birthday of mother, prefecture, municipality, multiplicity of birth, birth order for multiple births, and infant mortality caused by diseases were used in the mortality data. Information on birth characteristics were not available for mortality that occurred within 1 year from birth by external causes. Therefore, mortality caused by internal causes was used as infant mortality in this study.

### Data linkage

Two data linkages were conducted. The first data linkage was conducted between birth data and the Census to link the educational level to parents in the birth data. To identify births that resulted in infant mortality, the second data linkage was conducted between the birth data and mortality data.

Common information between the Census and birth data were used to link the two types of data; sex, birth year, birth month, prefecture, municipality, and nationality were used. However, too many men or women have become candidates for parents in this case. Therefore, two restrictions in the linkage were added, as was conducted in a previous study [19]. First, in the Census data, only married men and women were included to avoid matching non-married persons with parents of a legitimate child. In addition, only those who reside in a same household were considered eligible men and women for parents. Deterministic data linkage was conducted for each year for birth data, and only one-to-one matching pairs between the Census and birth data were used in the statistical analysis. However, for multiple births (twins, triplets and quadruplets, and et al.), multiple birth data need to be matched with one couple in the Census. Therefore, many-to-one matching between the Census and birth data were allowed only for those multiple births.

In data linkage between the birth data and infant mortality data, sex, birthday of infant, the birthday of mother, prefecture, municipality, multiplicity of birth, and birth order were used, as was used in a previous study [6]. Deterministic data linkage was conducted, and only one-to-one matching pairs between the two data were used in the statistical analysis. From the matched birth data, we extracted birth data which were matched with the Census data in the first data linkage.

### Statistical analysis

For the educational level, elementary school graduates were included in the category of junior high school graduates because education through junior high school is compulsory in Japan and the number was relatively small as a type of graduates. In addition, graduates from graduate schools were classified in this study as university graduates. Therefore, four educational levels were analyzed: “junior high school,” “high school,” “technical school or junior college,” and “university.” Maternal age was categorized per 5-year age groups, and parity was divided into primiparous and multiparous. In addition, birthweight, and gestational age were also grouped when tallying the data.

We tallied the number of births with and without infant mortality for each of the attributes. A multivariate logistic regression model was used to investigate an association between parental educational level and infant mortality. Infant’s sex, maternal age, multiplicity of births, parity, and household occupation were adjusted in the regression analysis. Adjusted odds ratio, 95% confidence interval (CI), and p-value were calculated for each attribute. Two-sided test was used, and p < 0.05 was considered as statistically significant.

Complete-case analysis was performed to deal with missing data. Sensitivity analysis was conducted using multiple imputations [21]. All the analyzes were conducted using R 4.1.3 [22]. The results shown in this study are based on the analysis by the authors using the data obtained from the Ministries, and those are different from statistics published by the Ministries. This study was approved by an ethical committee of Kyushu University (No. 22221–00). In addition, informed consent is not needed for this study because we used the Vital Statistics and Census data that were provided from the Ministries on the basis of the Statistics Act in Japan, and it was waived from the ethical committee.

## Results

**Fig 1:**
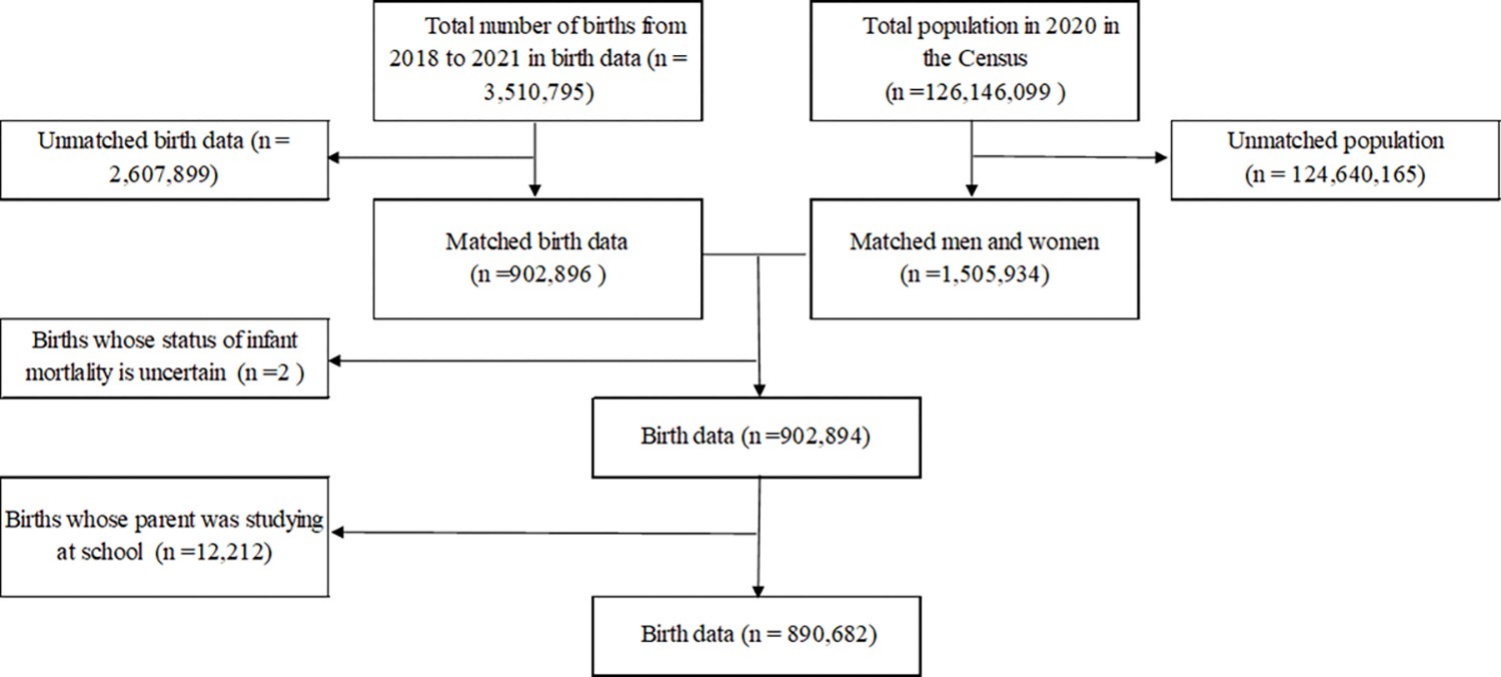
Result of data linkage between birth data and the Census data.

Fig 1 shows the results of data linkage between birth data and the Census data. Data on 3,510,795 births and 126,146,099 persons were used for data linkage, and 902,896 births were linked with the Census data. Finally, data on 890,682 births were analyzed.

**Fig 2:**
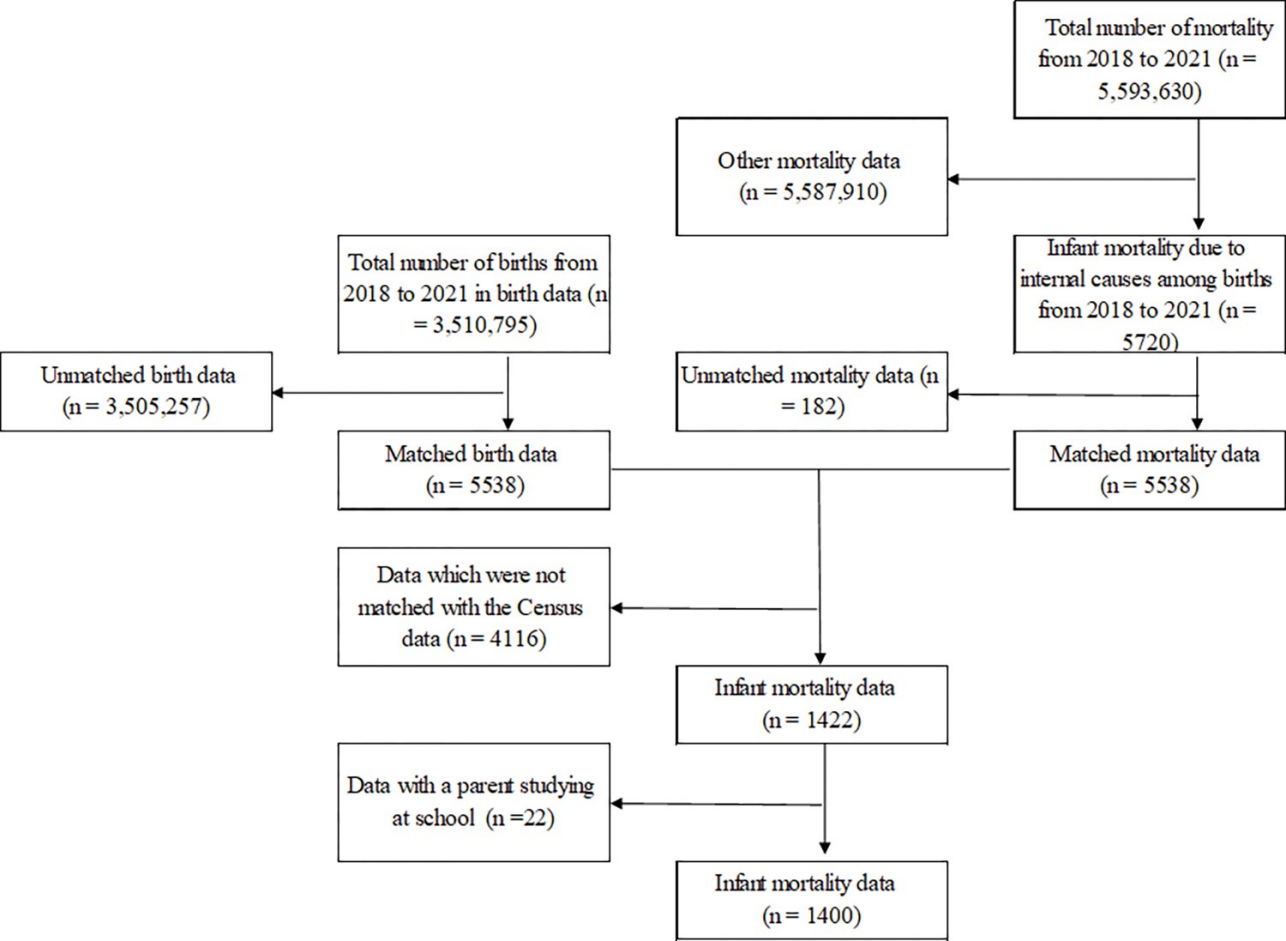
Result of data linkage between birth data and mortality data.

Fig 2 shows the results of data linkage between birth data and mortality data. Birth data and 5720 cases of infant mortality were used for linkage, resulting in 5538 matched cases. After restricting the infant mortality data to births which were matched with the Census data, data on 1400 infant mortality were analyzed.

Table 1 shows the number and proportion of each attribute for births with and without infant mortality. The proportion of junior high school or high school graduates was higher among fathers and mothers for births with infant mortality than that among those for births without infant mortality, and an opposite result was observed for the proportion of university graduates.

**Table 1:** Number and proportion of each attribute for births with and without infant mortality.

	Births without infant mortality	Births with infant mortality
	n (%)	n (%)
Total	889282 (100.0)	1400 (100.0)
Gender		
Female	433779 (48.8)	638 (45.6)
Male	455503 (51.2)	762 (54.4)
Maternal age group		
19 years or less	6542 (0.7)	22 (1.6)
20–24 years	108406 (12.2)	205 (14.6)
25–29 years	259235 (29.2)	323 (23.1)
30–34 years	287989 (32.4)	371 (26.5)
35–39 years	179755 (20.2)	346 (24.7)
40 years or more	47355 (5.3)	133 (9.5)
Parity		
Primiparous	406686 (45.7)	561 (40.1)
Multiparous	482596 (54.3)	839 (59.9)
Multiplicity		
Singleton	871398 (98.0)	1280 (91.4)
Multiple	17884 (2.0)	120 (8.6)
Household occupation		
Farmer	16986 (1.9)	39 (2.8)
Self-employed	71404 (8.0)	118 (8.4)
Full-time worker 1	309557 (34.8)	528 (37.7)
Full-time worker 2	376354 (42.3)	508 (36.3)
Other occupations	81327 (9.1)	130 (9.3)
Unemployed	7215 (0.8)	23 (1.6)
Missing	26439 (3.0)	54 (3.9)
Paternal educational level		
Junior high school	52904 (5.9)	105 (7.5)
High school	295419 (33.2)	502 (35.9)
Technical school or junior college	111342 (12.5)	177 (12.6)
University or graduate school	302550 (34.0)	401 (28.6)
Missing	127067 (14.3)	215 (15.4)
Maternal educational level		
Junior high school	38729 (4.4)	92 (6.6)
High school	279984 (31.5)	493 (35.2)
Technical school or junior college	218259 (24.5)	315 (22.5)
University or graduate school	226784 (25.5)	289 (20.6)
Missing	125526 (14.1)	211 (15.1)
Gestational age		
Preterm birth	49324 (5.5)	683 (48.8)
Term birth	839832 (94.4)	715 (51.1)
Missing	126 (0.0)	2 (0.1)
Birthweight		
< 1500 g	6290 (0.7)	450 (32.1)
1500–2500 g	74133 (8.3)	376 (26.9)
> = 2,500g	808763 (90.9)	570 (40.7)
Missing	96 (0.0)	4 (0.3)

Table 2 shows the results of logistic regression analysis investigating an association between parental educational level and infant mortality. Regression analysis results showed that mothers who finished junior high school or high school were significantly and positively associated with infant mortality, compared with those who were university graduates. Odds ratios also shows a positive association for fathers who finished junior high school or high school with infant mortality, but not significantly different from those of fathers who were university graduates.

**Table 2:** Result of logistic regression analysis investigating an association between parental educational level and infant mortality.

	Adjusted odds ratio (95%CI)	p-value
Gender		
Female	Reference	
Male	1.153 (1.026, 1.296)	0.017
Maternal age group		
19 years or less	2.037 (1.245, 3.332)	0.005
20–24 years	1.276 (1.041, 1.564)	0.019
25–29 years	0.948 (0.803, 1.119)	0.524
30–34 years	Reference	
35–39 years	1.386 (1.179, 1.631)	<0.001
40 years or more	2.261 (1.829, 2.796)	<0.001
Parity		
Primiparous	Reference	
Multiparous	1.136 (1.003, 1.287)	0.044
Multiplicity		
Singleton	Reference	
Multiple	4.640 (3.775, 5.704)	<0.001
Household occupation		
Farmer	1.460 (1.033, 2.064)	0.032
Self-employed	1.053 (0.836, 1.327)	0.661
Full-time worker 1	1.147 (1.002, 1.313)	0.047
Full-time worker 2	Reference	
Other occupations	1.111 (0.900, 1.371)	0.327
Unemployed	1.891 (1.144, 3.125)	0.013
Paternal educational level		
Junior high school	1.228 (0.965, 1.562)	0.095
High school	1.133 (0.978, 1.313)	0.097
Technical school or junior college	1.125 (0.933, 1.355)	0.217
University or graduate school	Reference	
Maternal educational level		
Junior high school	1.514 (1.157, 1.981)	0.002
High school	1.247 (1.057, 1.470)	0.009
Technical school or junior college	1.057 (0.893, 1.251)	0.519
University or graduate school	Reference	

CI; confidence interval

S1 Table shows the results of logistic regression analysis using multiple imputations. A similar result was obtained in a complete-case analysis; mothers who finished junior high school or high school were statistically significantly and positively associated with infant mortality, compared with those who were university graduates.

## Discussion

This study investigated the association between parental educational level and infant mortality in Japan. Our results showed that a low educational level for mothers was positively associated with infant mortality. The result is similar to previous studies in other countries, which showed that infant mortality rate was higher in mothers with lower educational level [13, 14], and it is consistent with the worldwide trend [16]. Although we did not identify reasons for the association in this study, we discuss possible reasons for the association and an implication of this study. Specifically, associations between smoking prevalence and educational level, between access to medical care and educational level, and between adverse birth outcomes and educational level are discussed.

The low educational level of mothers was associated with infant mortality possibly due to other adverse birth outcomes. In Japan, lower parental educational level is associated with a higher preterm birth rate [19], and parental educational level of ≥16 years reduced risk of term-small-for-gestational-age (term-SGA) [18]. Preterm birth and SGA are risk factors for infant mortality [8], and it is considered that those are intermediates. Educational disparity in preterm birth is larger in mothers than that in fathers in Japan [19], and it is one possible reason why a significant association was shown only for mothers but not for fathers.

It is considered that some factors are becoming intermediates between lower educational level and the adverse birth outcome. Smoking is considered a factor for the association between the adverse birth outcomes and educational level because a lower educational level is associated with a higher smoking rate in Japan [23] and smoking is a risk factor for the adverse birth outcomes [24, 25]. Although there is a result that paternal smoking also affects preterm birth [26], maternal smoking rather than paternal smoking is associated more with adverse birth outcomes [25, 27].

Access to medical facilities can be another reason for the association between infant mortality and educational level. Infant mortality rate is particularly high in jobless households in Japan, and decreased access to medical facilities was pointed out as a possible factor for this phenomenon [10]. In Japan, low-income individuals have poorer access to outpatient care [28]. In addition, greater education for parents provides social networks that is related to safer child environments [29, 30], and it might also be related to the educational disparity. Moreover, an ecological study showed that percentages of high school enrolment was negatively associated with inadequate prenatal care use among regions in Japan [31]. Therefore, it is possible that mothers with lower educational level have lesser access to prenatal care or medical facilities in Japan.

Although an association between infant mortality and parental socioeconomic status was not conclusive in Japan from previous studies [9, 11], results of this study implies that infant mortality is associated with maternal socioeconomic status. To decrease a disparity in infant mortality, it is important to prevent a higher rate of adverse birth outcomes, such as preterm birth and SGA, among mothers with lower educational level or socioeconomic status. Amelioration of health behavior for parents can lead to a decrease in disparity in adverse birth outcomes, and it might lead to a decrease in disparity in infant mortality. In addition, financial assistance for mothers with lower socioeconomic status might also alleviate the disparity if low-income or educational level is a cause of lower use of medical services. It is known that an increase in public subsidies for prenatal care had a positive effect on reduction of low birth weight rate in Okinawa in Japan by increasing number of prenatal care visits for mothers [32], and a similar result of financial aid for prenatal care was also observed in the Unites States [33]. In contrast, studies for an effect of financial aid is limited in Japan, and a further study is needed for investigating the association. Moreover, it is meaningful to investigate differences in health statuses and behaviors of mothers depending on maternal educational level in future studies.

The study had limitations including data linkage. First, our results were based on data linkage. Therefore, it is possible that some errors in matching occurred in the data linkage.

In contrast, it is prohibited for citizens to write false information on those national surveys in Japan, and it is considered that data with were used were basically accurate. If the information in the data were accurate, parental educational levels could be correctly attached to birth data because one-to-one matching pairs were used in the analysis. Second, there were many birth data that could not be matched with the Census data, and the analysis result of this study is not based on all births in Japan. Third, infant mortality caused by external causes was not included in this study because the data were not available in the Vital Statistics. Fourth, we used the Vital Statistics from 2018 to 2021, while the Census data in 2020 were used. Therefore, it is possible that there were some mothers or fathers who had not attained their highest educational attainment at the time of giving birth to a child.

## Conclusion

In this study, we investigated the association between parental educational level and infant mortality using the Vital Statistics and the Census in Japan. As a result, the proportion of junior high school or high school graduates was higher among fathers and mothers for births with infant mortality compared with that among those for births without infant mortality; in contrast, the proportion of university graduates was lower for births with infant mortality than those without infant mortality. Regression analysis showed that mothers with junior high school or high school graduates were significantly and positively associated with infant mortality compared with those with university graduates. As a conclusion, lower educational level in mothers was positively associated with infant mortality, and it was shown that a difference in infant mortality depending on parental educational level existed in Japan.

## Supporting information

S1 Table Result of logistic regression analysis investigating an association between parental educational level and infant mortality using multiple imputation. (PDF)
