# Supplementary material for: Association between infant mortality and parental educational level: An analysis of data from Vital Statistics and Census in Japan
Source: PLoS One. 2023 Jun 14;18(6):e0286530. doi: 10.1371/journal.pone.0286530 (PMC10266618; doi:10.1371/journal.pone.0286530)
Supplement: S1 Table — (PDF) [file pone.0286530.s001.pdf]

TableS1. Result of logistic regression analysis investigating an association between parental educational level and infant mortality using multiple imputation.

	Adjusted odds ratio (95%CI)	p-value
Gender		
Female	Reference	
Male	1.144 (1.029, 1.271)	0.013
Maternal age group		
19 years or less	2.264 (1.448, 3.540)	<0.001
20-24 years	1.369 (1.143, 1.640)	<0.001
25-29 years	0.968 (0.832, 1.125)	0.668
30-34 years	Reference	
35-39 years	1.470 (1.269, 1.703)	<0.001
40 years or more	2.138 (1.752, 2.608)	<0.001
Parity		
Primiparous	Reference	
Multiparous	1.132 (1.012, 1.267)	0.03
Multiplicity		
Singleton	Reference	
Multiple	4.484 (3.712, 5.418)	<0.001
Household occupation		
Farmer	1.533 (1.101, 2.134)	0.011
Self-employed	1.107 (0.902, 1.359)	0.331
Full-time worker 1	1.182 (1.042, 1.342)	0.009
Full-time worker 2	Reference	
Other occupations	1.142 (0.941, 1.386)	0.179
Unemployed	2.030 (1.331, 3.097)	0.001
Paternal educational level		
Junior high school	1.163 (0.894, 1.513)	0.26
High school	1.140 (0.969, 1.340)	0.113
Technical school or junior college	1.136 (0.940, 1.373)	0.188
University or graduate school	Reference	
Maternal educational level		
Junior high school	1.435 (1.100, 1.872)	0.008
High school	1.194 (1.015, 1.405)	0.033
Technical school or junior college	1.046 (0.881, 1.243)	0.606
University or graduate school	Reference	

CI; confidence interval
